# Single channel medical images enhancement using fractional derivatives

**DOI:** 10.1371/journal.pone.0319990

**Published:** 2025-05-21

**Authors:** Anand Singh, Mohammad Sajid, Naveen Kumar Tiwari, Anurag Shukla

**Affiliations:** 1 Department of Electrical Engineering (Cyber Physical Systems), Indian Institute of Technology Jodhpur, Jodhpur, India; 2 Department of Mechanical Engineering, College of Engineering, Qassim University, Buraydah, Saudi Arabia; 3 Department of Computer Science and Engineering, Rajkiya Engineering College Kannauj, Kannauj, India; 4 Department of Applied Sciences (Mathematics), Rajkiya Engineering College Kannauj, Uttar Pradesh, India; Al-Nahrain University, IRAQ

## Abstract

The current research uses the Grünwald–Letnikov (GL) fractional differential mask to improve satellite and medical images. One of the important image enhancement methods in digital image processing is texture enhancement. A fractional differential-based two-dimensional discrete gradient operator is based on the definition of Grünwald–Letnikov (GL) interpretation of fractional calculus, which is extended from a one-dimensional operator through the analysis of its spectrum to improve the image texture. Which then extracts more subtle texture information, and gets around the lack of a classical gradient operator. Based on the GL fractional differential, an approximate two-dimensional isotropic gradient operator mask was created using the GL fractional derivative, the technique generates 3×3 and 5×5 pixel-sized masks that preserve the correlation between neighboring pixels. The strength of the mask, which was a variable and non-linear filter, could be changed by varying the intensity factor to enhance the image. Experimental results show that the operator may emphasize the texture and obtain more complex information. Compared to the conventional classical methods, the suggested way has an excellent promotional effect on texture enhancement compared to the previous method on grayscale images.

## 1 Introduction

Texture enhancement is crucial in image processing applications, ranging from medical image analysis to pattern recognition. Traditional techniques, such as histogram equalization, though popular, often lead to image over-enhancement. Recent research indicates that fractional calculus offers a promising alternative.

Various methods in fractional calculus have shown potential in image processing. The Grunwald-Letnikov approach, for instance, is known for its straightforward numerical implementation, making it suitable for texture enhancement tasks. This method offers a clear interpretation of fractional derivatives, which can be advantageous in capturing fine image details.

The Atangana-Baleanu derivative, applied in the Caputo sense, introduces a non-local and non-singular kernel. This characteristic allows for better handling of image textures and edges, ensuring that crucial image features are preserved during enhancement. Its application in image enhancement demonstrates a significant improvement in maintaining the integrity of medical images.

Another notable method is the Riemann-Liouville (RL) derivative combined with the Mittag-Leffler function. This approach leverages the memory effect of fractional derivatives, which is particularly useful in medical imaging, where historical data plays a crucial role in accurate diagnosis. By integrating the Mittag-Leffler function, this method enhances the fractional derivative’s adaptability, resulting in more precise texture enhancement.

The Taufik Atangana method also introduces a new perspective by integrating fractal-fractional operators. This method accounts for the fractal nature of image textures, enabling more detailed and nuanced enhancement. The Taufik Atangana approach is particularly effective in dealing with complex textures and intricate patterns, providing more detail preservation in medical images.

In summary, fractional calculus generalizes derivatives and integrals to non-integer orders, providing a flexible framework for image processing. Fractional derivatives are particularly effective in preserving fine details and reducing noise due to their non-local and non-singular properties. This adaptability enables better capture of intricate textures and smoother transitions, crucial for enhancing medical images where detail preservation is paramount. The ability to dynamically adjust the fractional order further improves adaptability by tailoring enhancement to specific image features.

Our approach addresses the limitations of existing methods by introducing a novel technique that leverages fractional derivatives. This technique allows for adaptive enhancement based on the specific features of each image in a single channel. Additionally, we employ non-singular masks to target desired details while minimizing unwanted artifacts precisely, thus significantly improving upon traditional singular masks commonly used in image enhancement.

Building on the success of fractional calculus in emphasizing textural features [1], our method diverges from prior approaches that use a fixed fractional order for the entire image. Instead, we propose a mask that dynamically adjusts the fractional order for each pixel, with a particular focus on enhancing textures in medical images. This innovation, combined with the GL fractional differential operator, overcomes the drawbacks of the RL operator and classical methods, paving the way for a more effective image enhancement technique.

The key contributions of this work are listed below:

1. A novel mask based on a modified GL and Atangana-Baleanu in the Caputo sense (ABC) derivative has been proposed to extract relevant features required for enhancement.
2. The framed mask is applied to the image to produce an enhanced image with improved edges and texture while preserving the smooth regions. The fractional order is determined separately for each of the regions.
3. When tested against the other fractional derivative-based methods, the proposed masks achieve better results without increasing computational requirements.

## 2 Literature survey

Many researchers have worked on these derivatives, some of the key works have been discussed below. For the goal of eliminating noise from an image, Ghanbari and Atangana *et al*. [2] expanded the Atangana-Baleanu fractional integral. They got outstanding results due to the effectiveness of the new fractional mask. Babu *et al*. [1] developed a new enhanced edge detection (EED) which is designed by integrating an image enhancement algorithm with edge detection techniques for low-contrast images. In another article, IDCP BFD algorithm [3], a bilateral fractional differentiation technique was proposed that improved an image’s edge details by using fractional differentials in both the spatial and intensity domains. They finally put the inversion procedure into practice. The experimental findings demonstrate the proposed method’s superiority to methods of relative comparison. Another method proposed by Nandal *et al*. [4] delivered an outcome from the experimental results which are presented as qualitative texture results and in tabular form. The results demonstrate that the suggested method outperforms traditional edge detection operators for any grayscale image. The Savitzky-Golay differentiator (DFOSGD) method [5] was also investigated in the image improvement application. The moving window in DFOSGD is in charge of calculating the desired fractional order’s fractional derivative for a given signal. This mask is used to improve dark and low-contrast photographs as well as to characterize the fractional derivative for an image. A Grünwald-Letnikov method suggested by Luo *et al*. [6] generates generic masks with eight directions and fractional calculus. Notably, it is essential to follow the filtering rule while performing fractional filtering of the image’s gray level values (intensity or brightness) in color space (hue, saturation, intensity, or brightness). Through the use of the gray-scale histogram and information entropy, they have investigated the quantitative examination of enhancement performance. Another method by Hu *et al*. [7] involves 3 × 3 and 5 × 5 pixel-sized masks that maintain the correlation between nearby pixels using the GL definition of the fractional derivative where the threshold is determined using a gradient. It is possible to divide an image into edge, texture, and smooth regions.

An image enhancement method proposed by He *et al*. [8] offers a fractional order picture denoising technique that can keep edges and significant edge features. Pu *et al*. [9] used the RL fractional differential-based method to enhance the texture and luminosity of the image. Motłoch *et al*. [10] proposed an improved, corrected version of the fractional derivative-based edge detection masks. Several alternative approximations of this derivative were used to implement the masks. The quality of image fusion did not significantly improve with the analyzed change of several approximations. The adaptive fractional differential method [11] offers a suggested AFDA method that produces better-enhancing results since it fully takes into account both global and local information in medical images in comparison to conventional image processing techniques. Kumar *et al*. [12] proposed a novel conformable fractional derivative-based nonlinear diffusion model for image denoising given in the article. As demonstrated by numerous fractional orders, this model is particularly effective at reducing the noise of degraded noisy images. Jalalinejad *et al*. [13] offers a fresh, highly adaptable definition of the Grünwald-Letnikov fractional derivative in their study. Which can be used in a variety of image-processing applications, including edge detection, picture enhancement, and medical diagnostics. Loverro [14] aims to introduce the reader to the fundamentals of fractional calculus while also whetting his or her desire for scientific and engineering applications. Despite the young age of this topic, the short selection of relevant problems shown here only represents a very small portion of what is now being researched. The rough set and fractional order differentiator method proposed by Zhang and Dai [15] uses rough set theory and Gaussian mixture model which are utilized in this segmentation procedure to group gray levels into various image layers. To further centralize the information, gray levels are compressed as well as filled in when they are blank. The depth image denoising algorithm by Huang *et al*. [16] studies the field of depth image processing introduced to the fractional calculus and a fractional integral denoising operator mask appropriate for depth image processing is defined and built. Simulation tests, for the Redwood dataset, were used to validate the suggested depth image denoising algorithm..

Amoako-Yirenkyi *et al*. [17] suggested a fractional gradient descent method for neural network backpropagation (BP) training. Specifically, the fractional-order gradient of the error defined as the conventional quadratic energy function is evaluated using the Caputo derivative. Azarang *et al*. [18] used the CS framework to provide a novel pan-sharpening technique. Eight partial masks are used to create the superimposed mask in eight different directions. The high-frequency information can be more effectively injected into the low-resolution MS image using the suggested method.

Jones *et al*. [19] suggested fractional order edge detection filters to assess the outcomes. After performing a visual examination of edge detection, they discovered that the suggested filters could modify the level of edge detection by varying the fractional differential order, which also turns out to be a good localization and sharper edge detection method. Wang *et al*. [20] proposed a technique that enables edge detection of a noisy image. Mask construction offers the ability to consistently and accurately identify edges in various directions.

Most of these new fractional masks are presented by Ghanbari and Atangana *et al*. [21] in their work for the first time. Furthermore, the proposed fractional masks practically use the same number of calculations as the conventional ones in terms of computational cost. To have a broad understanding of the idea of fractional derivatives, this study has also provided a list of expressions. Teodoro *et al*. [22] proposed several equations that do not use the term fractional. The piecewise gamma-corrected method by Singh *et al*. [23] used a modified Cuckoo Search (MCS) optimization model using a newly designed Gray-Level Co-occurrence Matrix (GLCM) features-based function that is influenced by the golden ratio. Lavín-Delgado1 *et al*. [24] had the primary accomplishment of their study which is, the creation of a Caputo-Fabrizio derivative-based fractional-order edge detector. The proposed innovative fractional mask without a singular kernel outperforms other approaches described in the literature in terms of visual and quantitative analysis.

Yang *et al*. [25] used fractional calculus definition (GL, RL, and Caputo) to generalize the ordinary differentiation and integration to fractional order (arbitrary order). A new mask based on the Atangana-Beleanu fractional operator is suggested in this work. Convolutional Atangana-Baleanu method [26] similar to integer-order derivative mask known as the Canny edge detector has not only the same complexity, but it also has the added benefit of being able to characterize non-local and non-singular edge maps more effectively. Auto-correlation function method proposed by Hemalatha *et al*. [27] uses a filter and its suitability for texture improvement is examined while taking into account the fundamental definition and application of the GL-based fractional differential operator. The threshold is determined using a gradient, allowing the input image to be divided into edge, texture, and smooth regions. Wadhwa and Bhardwaj [28] suggested that the order of the fractional derivative should be chosen individually for each pixel in each zone, and the framed mask is then applied to the input image to create a better image. Ortigueira *et al*. [29] provide a new construction of a fractional-based convolution mask that is significantly more noise-resistant while having equal complexity *O*(*mnlog*(*mn*)) as the conventional gradient operators. Due to dealing with the most typical formulations, the author of this article [30] refers to those who we believe are most significant in the field.

Liu *et al*. [31] investigated the use of symmetry analysis to explore various time-space fractional integrable equations including the KdV-type equations, Burgers equations, and unsteady Euler equations of gas dynamics. Liu *et al*. [32] proposed that fractional calculus is an essential tool for describing nonlinear phenomena in applied sciences. Their work specifically investigates the Korteweg-de Vries (KdV)-like equation, which models shallow water wave evolution, using the Riemann-Liouville space-time fractional derivative

Some recent advancements in mathematical modeling have emphasized the significance of fractional-order differential equations in capturing complex biological and physical phenomena. Sweilam *et al*. [33] explored the application of the Ψ-Caputo derivative and Mittag-Leffler laws to develop a novel crossover mathematical model for breast cancer, offering improved numerical treatments. Similarly, the comprehensive work by Radwan *et al*. [34] highlighted the utility of fractional-order modeling across dynamic systems, with applications spanning optimization, signal processing, and control. In a related context, Sweilam *et al*. [35] proposed a crossover dynamics model for monkeypox using fractional differential equations, further demonstrating the versatility of the Ψ-Caputo derivative in addressing emerging epidemiological challenges. These studies collectively underline the growing relevance of fractional calculus in modeling and analyzing dynamic systems.

## 3 Proposed method

A 3×3 and 5×5 differential mask order is proposed in this research article This takes into account the association between nearby pixels. The following are the work’s highlights:

1. To create a mask that maintains the correlation of the nearby pixels, the technique applies the GL and ABC concept of fractional derivative.
2. It applies fractional GL-integrals in both the x and y directions, effectively capturing fine details and textures in images with high accuracy.
3. The derived coefficients ($\rho_{0},\rho_{1},\rho_{2},\rho_{3}$) facilitate a flexible mechanism for multi-scale feature extraction and image enhancement, ensuring efficient and scalable performance.
4. The framed mask is applied to the image to produce an enhanced image with accentuated edges and texture while preserving the smooth parts. The fractional order is determined separately for each of the three regions.

Our method differs significantly from earlier work in the sense that we apply a mask that replaces the variable fractional order corresponding to each pixel in the image. In contrast to Wadhwa and Bhardwaj [28], we have found two criteria to divide an image into three sections using the mean of the gradient image, which is much more straightforward and understandable. The workflow is depicted in 1

**Fig 1:**
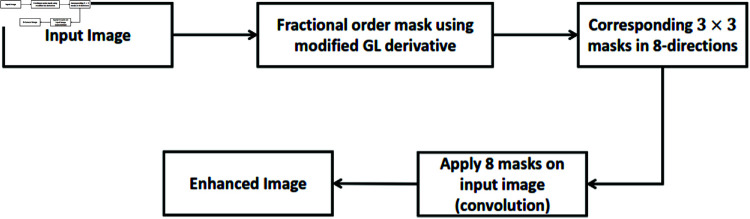
Workflow of proposed method.

The available literature sows that there is no unique definition of the fractional derivatives. There are four different classical definitions which are given as Grünwald-Letnikov (GL), Riemann-Liouville (RL), Caputo, and Atangana-Baleanu in Caputo and RL sense [1,2,28]. The literature also makes it obvious that the GL definition is best suited for signal and image processing applications.

$$f\;\prime (t)=\lim_{h\to 0}\frac{f(t)-f(t-h)}{h},$$ (1)

$$f\;\prime \prime (t)=\lim_{h\to 0}\frac{f(t)-2f(t-h)+f(t-2h)}{h^{2}},$$ (2)

$$f\;\prime \prime \prime (t)=\lim_{h\to 0}\frac{f(t)-3f(t-h)+3f(t-2h)-f(t-3h)}{h^{3}},$$ (3)

$$f\;\prime \prime \prime \prime (t)=\lim_{h\to 0}\frac{f(t)-4f(t-h)+6f(t-2h)-4f(t-3h)+f(t-4h)}{h^{4}}.$$ (4)

and using the concept of mathematical induction, the formula for ${\text{n}}^{\text{th}}$ order derivative is given as

f(n)(t)=limh→0∑j=0n(−1)j(Nj)(t−jh)hn,j∈ℕ (5)

The Grunwald-Letnikov definition for a fractional-order derivative is given by:

Dt(α)f(t)=limh→01hα∑k=0⌊th⌋(−1)k(αk)f(t−kh), (6)

where α is the fractional order, *h* is the step size, and (αk) is the binomial coefficient generalized to real numbers.

The definition provided by Atangana and Baleanu in the Caputo sense is one of the most widely used definitions for a fractional-order derivative (ABC).

$$D_{t}^{\left(n\right)}\kappa (t)=\frac{M(n)}{1-n}\int_{0}^{t}E_{(}n)\left[-n\frac{(t-\tau {)}^{n}}{1-n}\right]\kappa (\tau )\;d\tau .$$ (7)

The definition of this derivative uses a non-local, variable, and non-singular kernel, which is one of its standout features. The derivative also maintains a memory attribute that stores the details of the key function from the starting point until the desired time. Utilizing the Mittag-Leffler function of an index, which is described as follows, is a crucial aspect of this definition [21].

$$E_{\left(n\right)}(t)=\sum_{k=0}^{\infty }\frac{t^{k}}{\Gamma (nk+1)},n>0,$$ (8)

where *M*(.) is a normalization function that is utilized in this definition and is defined by:

$$M(n)=\frac{1-n+n}{\Gamma (n)}.$$ (9)

The equivalent definition for the fractional integral of Atangana-Baleanu of order for a function κ(t) is similarly defined as follows:

$$I_{t}^{\left(n\right)}\kappa (t)=\frac{1-n}{M(n)}\kappa (t)+\frac{n}{\Gamma (n)M(n)}\int_{0}^{t}\kappa (\tau ){\left(t-\tau \right)}^{\left(n-1\right)}\;d\tau$$ (10)

Numerous numerical techniques have been presented thus far to approximate these fractional operators utilizing various principles. We will use a variety of strategies to create new masks in this section.

Therefore, the mask based on the GL approximation of the fractional integral in the Atangana-Baleanu can be given as:

$$I_{GL}^{\left(n\right)}\kappa (t)=\frac{1-n}{M(n)}\kappa (t)+\frac{n}{\Gamma (n)M(n)}\int_{0}^{t}\kappa (\tau ){\left(t-\tau \right)}^{\left(n-1\right)}\;d\tau .$$ (11)

We now try to approximate the associated integral Grünwald-Letnikov up to *N* terms.

$$I_{GL}^{\left(n\right)}\kappa (t)\approx \int_{0}^{t}\frac{\kappa (\tau )}{{\left(t-\tau \right)}^{\left(n-1\right)}}\;d\tau =\lim_{h\to 0}h^{-n}[\kappa (t)+n\kappa (t-h)+\frac{\left(-n)(-n+1\right)}{2}\kappa (t-2h)\;+...+\frac{\tau (-n+1)}{k!(-n-N+1)}\kappa (t-Nh)]$$ (12)

We now try to approximate the associated integral Grünwald-Letnikov. This definition, which may be one of the most popular ones in discrete fractional calculus, has also been applied to image processing.

$$I_{GL}^{\left(n\right)}\kappa (t)\approx \frac{1-n}{M(n)}\kappa (t)+\frac{n}{M(n)}[\kappa (t)+n\kappa (t-h)+\frac{\left(n)(n-1\right)}{2}\kappa (t-2h)+\frac{n(1-n^{2})}{6}\kappa (t-3h)+\ldots ]$$ (13)

Fractional GL-integrals in the x and y directions are obtained according to the following equations:

$$I_{GL}^{x(n)}\kappa (x,y)\approx [\frac{1}{M(n)}\kappa (x,y)+\frac{n^{2}}{M(n)}\kappa (x-1,y)+\frac{n^{3}-n^{2}}{2M(n)}\kappa (x-2,y)+\frac{n^{2}-n^{4}}{6M(n)}\kappa (x-3,y)+...]$$ (14)

$$I_{GL}^{y(n)}\kappa (x,y)\approx [\frac{1}{M(n)}\kappa (x,y)+\frac{n^{2}}{M(n)}\kappa (x,y-1)+\frac{n^{3}-n^{2}}{2M(n)}\kappa (x,y-2)+\frac{n^{2}-n^{4}}{6M(n)}\kappa (x,y-3)+...]$$ (15)

The required coefficients for the method are computed in the following way:

$$\rho_{0}=\frac{1}{M(n)},\;\rho_{1}=\frac{n^{2}}{M(n)},\;\rho_{2}=\frac{n^{3}-n^{2}}{2M(n)},\;\rho_{3}=\frac{n^{2}-n^{4}}{6M(n)}.$$ (16)

Eight fractional differential masks, which include the directions of negative x-coordinate, negative y-coordinate, positive x-coordinate, positive y-coordinate, left downward, right upward, left upward, and right downward diagonals. They are designed to obtain the fractional order on the multiple symmetric directions and make the fractional order masks have the anti-rotation capability and given in Table 1. A scaled version of these masks has been given in Table 2

**Table 1:** Resultant mask 3×3 for all 8 directions.

**positive y-axis**			**upper right axis**			**positive x-axis**			**lower right axis**		
0	$\rho_{1}$	0	0	0	$\rho_{1}$	0	0	0	0	0	0
0	$\rho_{0}$	0	0	$\rho_{0}$	0	0	$\rho_{0}$	$\rho_{1}$	0	$\rho_{0}$	0
0	0	0	0	0	0	0	0	0	0	0	$\rho_{1}$
											
**negative y-axis**			**lower left axis**			**negative y-axis**			**upper left axis**		
0	0	0	0	0	0	0	0	0	$\rho_{1}$	0	0
0	$\rho_{0}$	0	0	$\rho_{0}$	0	$\rho_{1}$	$\rho_{0}$	0	0	$\rho_{0}$	0
0	$\rho_{1}$	0	$\rho_{1}$	0	0	0	0	0	0	0	0

**Table 2:** Resultant scaled masks of size 3x3.

**positive y-axis**			**upper right axis**			**positive x-axis**		
$\rho_{1}/3$	$\rho_{1}/3$	$\rho_{1}/3$	$\rho_{0}/5$	$\rho_{1}/3$	$\rho_{1}/3$	$\rho_{0}/5$	$\rho_{0}/5$	$\rho_{1}/3$
$\rho_{0}/5$	$\rho_{0}/5$	$\rho_{0}/5$	$\rho_{0}/5$	$\rho_{0}/5$	$\rho_{1}/3$	0	$\rho_{0}/5$	$\rho_{1}/3$
$\rho_{0}/5$	0	$\rho_{0}/5$	0	$\rho_{0}/5$	$\rho_{0}/5$	$\rho_{0}/5$	$\rho_{0}/5$	$\rho_{1}/3$
**lower right axis**			**negative y-axis**			**lower left axis**		
0	$\rho_{0}/5$	$\rho_{0}/5$	$\rho_{0}/5$	0	$\rho_{0}/5$	$\rho_{0}/5$	$\rho_{0}/5$	0
$\rho_{0}/5$	$\rho_{0}/5$	$\rho_{1}/3$	$\rho_{0}/5$	$\rho_{0}/5$	$\rho_{0}/5$	$\rho_{1}/3$	$\rho_{0}/5$	$\rho_{0}/5$
$\rho_{0}/5$	$\rho_{1}/3$	$\rho_{1}/3$	$\rho_{1}/3$	$\rho_{1}/3$	$\rho_{1}/3$	$\rho_{1}/3$	$\rho_{1}/3$	$\rho_{0}/5$

**negative y-axis**			**upper left axis**		
$\rho_{1}/3$	$\rho_{0}/5$	$\rho_{0}/5$	$\rho_{1}/3$	$\rho_{1}/3$	$\rho_{0}/5$
$\rho_{1}/3$	$\rho_{0}/5$	0	$\rho_{1}/3$	$\rho_{0}/5$	$\rho_{0}/5$
$\rho_{1}/3$	$\rho_{0}/5$	$\rho_{0}/5$	$\rho_{0}/5$	$\rho_{0}/5$	0

The structure in Table 3 is used to make a new fractional-order mask called a bi-directional mask [21]. This bidirectional mask ensures smoother transitions and finer detail restoration, making it superior for tasks requiring delicate contrast improvement and texture clarity.

**Table 3:** Resultant bi-directional masks of size 3x3.

**positive y-axis**			**upper right axis**		
$-\rho_{1}/3$	$-\rho_{1}/3$	$\rho_{1}/3$	$-\rho_{0}/5$	$-\rho_{1}/3$	$-\rho_{1}/3$
$-\rho_{0}/5$	0	$\rho_{0}/5$	$-\rho_{0}/5$	0	$\rho_{1}/3$
$-\rho_{0}/5$	$\rho_{0}/5$	$\rho_{0}/5$	$\rho_{0}/5$	$\rho_{0}/5$	$\rho_{0}/5$
**positive x-axis**			**lower right axis**		
$-\rho_{0}/5$	$-\rho_{1}/5$	$-\rho_{1}/5$	$\rho_{0}/5$	$-\rho_{1}/5$	$-\rho_{1}/5$
$\rho_{0}/5$	0	$-\rho_{1}/5$	$\rho_{0}/3$	0	$-\rho_{1}/5$
$\rho_{0}/3$	$\rho_{0}/3$	$\rho_{0}/3$	$\rho_{0}/3$	$\rho_{0}/3$	$-\rho_{0}/5$
**negative y-axis**			**lower left axis**		
$\rho_{0}/5$	$\rho_{0}/5$	$-\rho_{0}/5$	$\rho_{0}/5$	$\rho_{0}/5$	$\rho_{0}/5$
$\rho_{0}/5$	0	$-\rho_{0}/5$	$\rho_{1}/3$	0	$-\rho_{0}/5$
$\rho_{1}/3$	$-\rho_{1}/3$	$-\rho_{1}/3$	$-\rho_{1}/3$	$-\rho_{1}/3$	$-\rho_{0}/5$
**negative y-axis**			**upper left axis**		
$\rho_{1}/3$	$\rho_{0}/5$	$\rho_{0}/5$	$-\rho_{1}/3$	$\rho_{1}/3$	$\rho_{0}/5$
$-\rho_{1}/3$	0	$\rho_{0}/5$	$-\rho_{1}/3$	0	$\rho_{0}/5$
$-\rho_{1}/3$	$-\rho_{0}/5$	$-\rho_{0}/5$	$-\rho_{0}/5$	$-\rho_{0}/5$	$\rho_{0}/5$

**Table 4:** Resultant mask of size 5×5.

$-\rho_{1}/3$	$-2\rho_{1}/3$	$-2\rho_{0}/5-\rho_{1}/3$	$2\rho_{1}/3$	$\rho_{1}/3$
$-2\rho_{1}/3$	$-3\rho_{0}/5$	$-\rho_{0}$	$3\rho_{0}/5$	$2\rho_{1}/3$
$-2\rho_{0}/5-\rho_{1}/3$	$-\rho_{0}$	0	$\rho_{0}$	$2\rho_{0}/5+\rho_{1}/3$
$-2\rho_{1}/3$	$-3\rho_{0}/5$	$\rho_{0}$	$3\rho_{0}/5$	$2\rho_{1}/3$
$-\rho_{1}/3$	$-2\rho_{1}/3$	2ρ0/5+ρ1/3	2 ρ1/3	ρ1/3

Any pixel in an image has a strong relationship between its value and the values of its surrounding pixels. As a pixel’s distance from its neighbors increases, this dependence diminishes. Five pixels are situated one pixel away from the central pixel. The weight is distributed evenly among these neighbors because the value of the central pixel depends equally on each of its five surrounding pixels. Similar to this, 3 pixels are 2 pixels from the center.

By considering multiple orientations—specifically eight directions at angles of (0^∘^,45^∘^,90^∘^,135^∘^,180^∘^,225^∘^,270^∘^,315^∘^,360^∘^) these masks provide more precise control over image details compared to traditional methods. Once we have obtained the masks for these eight directions, we ultimately combine them to create the 5×5 mask shown in Table 4. As a result, it is easier for the intensity values of the generated image to fall inside the [0,255]range, ensuring that the enhanced image remains within standard pixel intensity limits. This bidirectional approach allows for enhanced edge sharpening and noise reduction by selectively processing pixel intensity in multiple directions, ensuring smoother transitions and finer detail restoration. Consequently, it is superior for tasks requiring delicate contrast improvement and texture clarity.

## 4 Performance metrics

We used the standard metrics *i.e.*, peak signal-to-noise ratio (PSNR) and structural similarity index (SSIM) which are described below and support the performance of the suggested mask.

### 4.1 PSNR: Peak signal-to-noise ratio for image restoration

The Peak Signal-to-Noise Ratio (PSNR) is a widely used objective metric in image processing, particularly in image restoration tasks, to assess the quality of reconstructed images. PSNR measures the difference between the reference (original) image and the restored image by computing the ratio between the maximum possible pixel value (signal) and the noise introduced by the restoration process. It is commonly used because of its simplicity and ability to quickly quantify the error in image reconstruction.

PSNR is typically measured in decibels (dB), with higher PSNR values indicating better restoration quality and lower noise levels. While PSNR is widely adopted, it does not necessarily correlate with human visual perception. For this reason, metrics like the Structural Similarity Index (SSIM) are often used alongside PSNR to provide a more comprehensive assessment of image quality.

The PSNR between a reference image *I*_*ref*_ and a restored image *I*_*res*_ is defined as:

$$PSNR=10\cdot \log_{10}\left(\frac{MAX_{I}^{2}}{MSE}\right).$$ (17)

Where *MAX*_*I*_ is the maximum possible pixel value of the image. For 8-bit images, this value is typically 255. *MSE* stands for Mean Squared Error, which quantifies the average squared differences between the pixel values of the reference and restored images.

To compute PSNR, we first need to calculate the Mean Squared Error (MSE) between the reference and restored images. MSE is given by:

$$MSE=\frac{1}{m\cdot n}\sum_{i=1}^{m}\sum_{j=1}^{n}{\left[I_{ref}(i,j)-I_{res}(i,j)\right]}^{2}.$$ (18)

Where *I*_*ref*_(*i*,*j*) and *I*_*res*_(*i*,*j*) represent the pixel values at position *(i,j)* in the reference and restored images, respectively. *m* and *n* are the dimensions of the image (height and width, respectively).

MSE represents the average of the squared differences between corresponding pixel values in the two images. A smaller MSE indicates that the restored image is closer to the reference image, resulting in a higher PSNR value.

### 4.2 SSIM: Structural similarity index

The Structural Similarity Index (SSIM) is a widely used perceptual metric in image processing, particularly in image restoration tasks. Unlike traditional metrics like Mean Squared Error (MSE) or Peak Signal-to-Noise Ratio (PSNR), which focus on pixel-wise differences, SSIM considers structural information and aligns more closely with human visual perception. SSIM evaluates the similarity between a restored image and a reference image based on three perceptual components: luminance, contrast, and structure. These components are combined into a single score to assess the quality of the restored image.

SSIM is defined in the range of [−1,1], with a value of 1 indicating perfect structural similarity between the two images and lower values representing degradation in visual quality.

The SSIM index is computed based on three distinct components:

- **Luminance**: Measures the difference in brightness between the reference and restored images.
- **Contrast**: Evaluates the difference in intensity variation between the two images.
- **Structure**: Assesses the correlation between the structures of the reference and restored images.

These components are calculated over small windows in the images (typically 8 × 8 or 11 × 11 pixel patches), and the results are averaged over the entire image to obtain the overall SSIM score.

The SSIM between two image patches *x* and *y* is defined as:

$$SSIM(x,y)={\left[l(x,y)\right]}^{\alpha }\cdot {\left[c(x,y)\right]}^{\beta }\cdot {\left[s(x,y)\right]}^{\gamma }$$ (19)

Where l ( x, y ) is the luminance comparison function. c ( x , y ) is the contrast comparison function. s ( x , y ) is the structure comparison function. α,β, and γ control the relative importance of each component, typically set to 1.

The luminance component compares the brightness of the two images:

$$l(x,y)=\frac{2\mu_{x}\mu_{y}+C_{1}}{\mu_{x}^{2}+\mu_{y}^{2}+C_{1}}$$ (20)

Where μ x and μ y are the mean intensities of image patches *x* and *y*, respectively. *C*_1_ is a small constant to avoid instability when the denominator is close to zero.

The contrast component assesses the intensity variations between the two images:

$$c(x,y)=\frac{2\sigma_{x}\sigma_{y}+C_{2}}{\sigma_{x}^{2}+\sigma_{y}^{2}+C_{2}}$$ (21)

Where σ x and μ y are the standard deviations (contrast) of patches *x* and *y*, respectively. *C*_2_ is a constant, similar to *C*_1_, to prevent division by zero.

The structure component captures the correlation between image structures:

$$s(x,y)=\frac{\sigma_{xy}+C_{3}}{\sigma_{x}\sigma_{y}+C_{3}}$$ (22)

Where σ x and μ y is the covariance between the image patches *x* and *y*. *C*_3_ is another constant (typically set to C_3_=C_2_/2).

By combining the luminance, contrast, and structure components, the complete SSIM index is given by:

$$SSIM(x,y)=\frac{\left(2\mu_{x}\mu_{y}+C_{1}\right)\left(2\sigma_{xy}+C_{2}\right)}{\left(\mu_{x}^{2}+\mu_{y}^{2}+C_{1}\right)\left(\sigma_{x}^{2}+\sigma_{y}^{2}+C_{2}\right)}$$ (23)

Here, the three components are multiplied together and simplified into one equation. The constants *C*_1_, *C*_2_, and *C*_3_ are introduced to ensure numerical stability.

In image restoration tasks, SSIM is used to evaluate the similarity between a degraded or noisy image and its restored version. SSIM provides a more accurate reflection of visual quality compared to pixel-wise metrics such as MSE. Higher SSIM scores indicate better restoration quality, with a score of 1 indicating perfect structural fidelity to the reference image.

## 5 Results and analysis

In this section, we compare the proposed fractional-order mask’s performance with that of the conventional, widely used methods for texture enhancement [10]. It can be used as a benchmark for assessing how well texture-enhanced operators operate.

A greater PSNR value indicates a higher image quality since the PSNR is a crucial determinant of image quality [10]. Here, a lower MSE value and a higher PSNR number determine better results. 2 and 3 report the PSNR and SSIM values with the corresponding images produced by fractional and integer order methods respectively. Additionally, Table 5 depicts PSNR and SSIM values with varying kernel sizes. SSIM value close to 1 indicates a higher image quality since the importance of SSIM in determining image quality [10].

**Fig 2:**
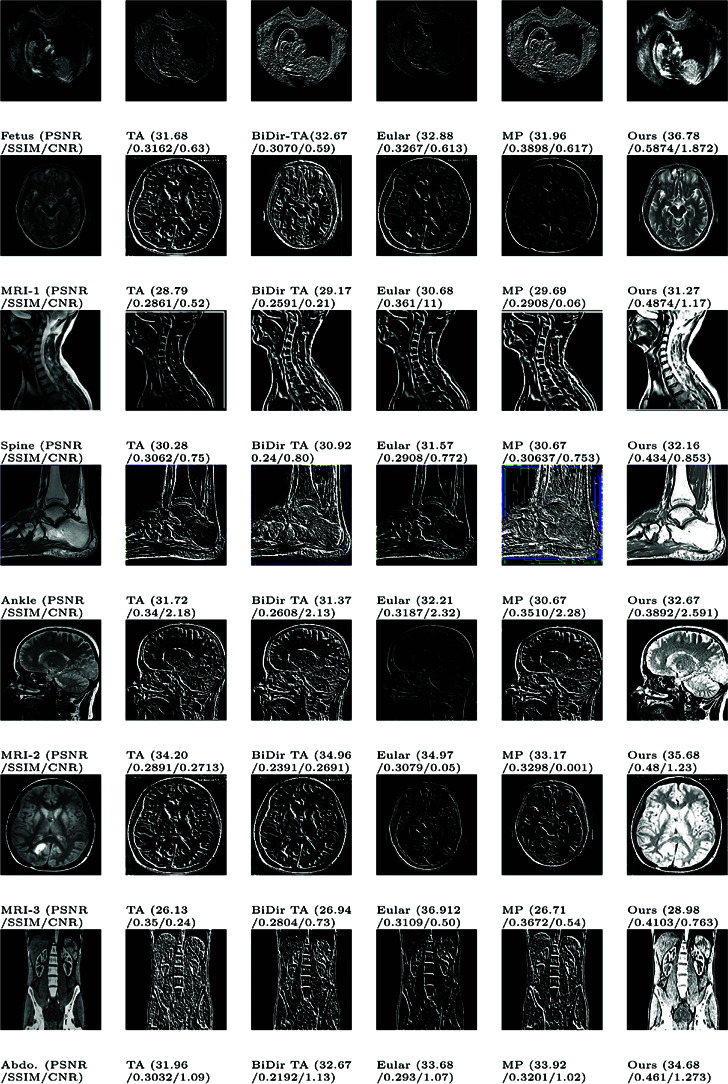
Visual results of various fractional order based methods obtained on test images.

**Fig 3:**
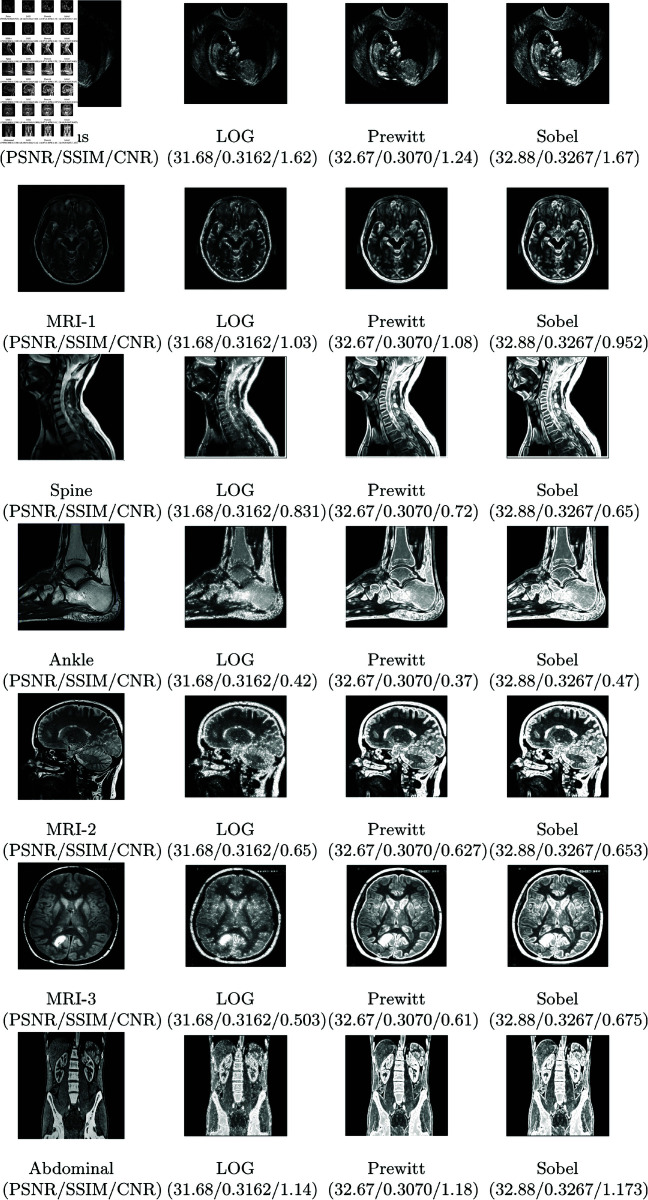
Visual results of various integer order based methods obtained on test images.

**Table 5:** PSNR and SSIM value comparisons for our method’s 3 × 3 5 × 5 mask.

Sr. No.	PSNR values of Sample Images		SSIM values of Sample Images	
	3 × 3 mask	5 × 5 mask	3 × 3 mask	5 × 5 mask
(1). Fetus	36.78	33.38	0.5874	0.4300
(2). MRI-1	31.27	27.81	0.4874	0.3202
(3). Spine	32.16	30.28	0.4340	0.2861
(4). Ankle	32.67	29.31	0.3892	0.3431
(5). MRI-2	35.68	33.91	0.4800	0.3678
(6). MRI-3	28.98	27.83	0.4103	0.3184
(7). Abdominal	34.68	33.37	0.4610	0.2904

Several performance metrics, including the noise level, processing time, error ratio, and edge continuity and relevancy have been considered. As a result, texture enhancement typically contains filtering steps to increase their robustness. An ideal robust texture enhancement should generate the same output for medical images. The suggested method retains the edges of the image while also improving the texture quality. The suggested method for improving image texture yields good results. The qualitative analysis of the offered method is one of the important factors for judging performance. We conducted a series of comparative experiments with various types of texture-enhancing algorithms to better understand the GL fractional differential-based algorithm’s power to enhance textures. The outcomes are displayed in Images (Fetus, MRI-1, Spine, Ankle, MRI-2, MRI-3, and Abdominal).

Fetus image [9] shows the impact of five different strategies on the improvement of ultrasound images. The initial image is noisy and has a low resolution because of the properties of ultrasonography. Although the histogram equalization method increases the object’s brightness, the processed image’s grayscale is diminished, and the local texture details vanish. The first-order approach draws edges nicely, but it severely mutes smooth areas and weak textures in the image. As a result, neither of these two approaches’ boosting results is sufficient. Image-7 (Abdominal) shows a sectional CT scan with drastically altered grayscale. Strong textures and smooth sections are drastically decreased by the first-order approach, making it impossible to view the entire image’s structure. Nonetheless, this method extracts a significant quantity of marginal information.

Images (MRI-1, Spine, MRI-2, MRI-3) [14] shows MRI images. It is demonstrated that the texture enhancement and the edges of the original photographs are evident. Similar to our proposed method, the previously proposed algorithm exhibits some improvements, but the grey area of the transitional area is unnatural, and the texture details of the image are not complete; as a result, the previously proposed algorithm does not improve the image as well as our suggested method.

The Ankle image shows the enhancement of an X-ray image. Our proposed method shows relatively better enhancement. However, the other proposed method is less effective and has marginal information.

The outcomes of the proposed method are reported in 4-5. It is worth noting that the first image of the ultrasound child with the high difference between the previous and proposed PSNR values is the best of all. 5 concludes that the suggested approach exhibits the highest SSIM, meaning it retains the target image’s edges up to the greatest extent among other methods. The majority of cases have the maximum PSNR value. It may be inferred by comparing the method described in this study with other widely used methods that these masks produce significantly better outcomes than these algorithms while requiring almost the same amount of processing. When outlining the edge of an image, these new masks can be a useful substitute for traditional masks.

**Fig 4:**
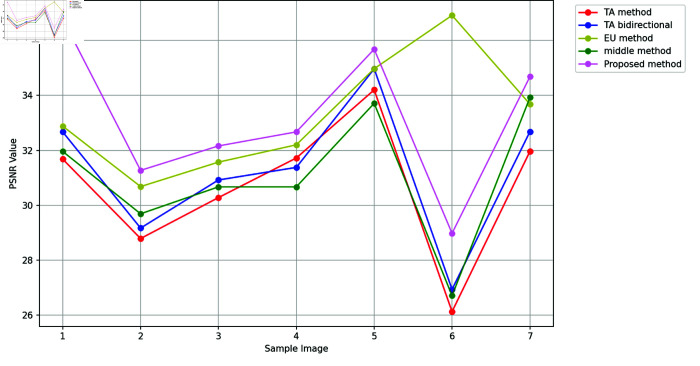
Comparison of texture enhancement with previous methods.

**Fig 5:**
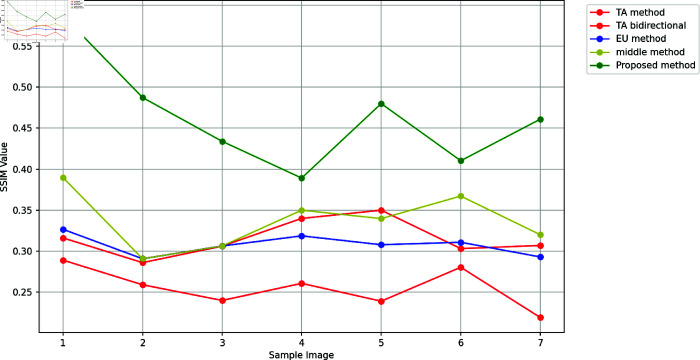
Comparison of texture enhancement with previous methods.

Table 6 provides a detailed comparison of the proposed method with other fractional-order approaches in terms of PSNR, SSIM, and CNR. The proposed method demonstrates superior performance overall, achieving a 4.2% to 8.3% improvement in PSNR and similar improvement in SSIM and CNR compared to existing methods. These results highlight the efficacy of the proposed approach in maintaining structural similarity and enhancing contrast in reconstructed images. There are few instances where integer order methods show comparable results, but they lag behind fractional order methods in a holistic comparison.

However, it is observed that the Euler approach outperforms the proposed method in terms of CNR for specific cases. This can be attributed to the inherent characteristics of the Euler method, which may enhance the edge and boundary contrast more effectively in certain high-frequency regions of the images. While this provides a localized improvement in contrast, the proposed method focuses on achieving a balanced enhancement across the entire image, resulting in a more consistent overall performance. Consequently, the proposed method ensures better preservation of fine details and structure, which is reflected in its superior PSNR and SSIM values.

**Table 6:** Quantitative comparison of the proposed work with other similar works. Best and second-best results are highlighted and underlined respectively.

Sr. No.	Method	PSNR	SSIM	CNR
1.	Toufik–Atangana (TA) approach [2]	30.96	0.3186	0.91
2.	Bidirectional Toufik–Atangana (TA) approach [2]	31.96	0.2585	0.77
3.	Euler approach [21]	33.13	0.3299	2.77
4.	Middle point (MP) approach[21]	31.54	0.3364	0.64
5.	LOG (Integer Order)	31.68	0.3162	1.14
6.	Prewitt (Integer Order)	32.67	0.3070	1.18
7.	Sobel (Integer Order)	32.88	0.3267	1.17
8.	Proposed approach	33.89	0.4642	1.25

### 5.1 Comparison of computational complexity

Fractional calculus methods, including Grunwald-Letnikov (GL) and Atangana-Baleanu in Caputo sense (ABC) derivatives, involve complex iterative operations and non-integer differentiation, which are computationally intensive compared to traditional methods like histogram equalization or Sobel edge detection. The proposed method’s use of dynamic fractional order adjustments and larger, multi-directional masks further adds complexity. In contrast, traditional techniques rely on fixed-size masks and simpler, uniform operations, resulting in lower computational demands.

### 5.2 Optimization techniques for real-time applications

To address computational challenges, parallel processing using GPUs can significantly accelerate the proposed method by handling convolution operations concurrently. Optimized algorithms, such as FFT-based approaches and sparse matrix representations, can further reduce computational and memory overhead. Approximation techniques for fractional derivatives and efficient adaptive thresholding minimize unnecessary calculations, enhancing processing speed.

### 5.3 Parameter selection and sensitivity analysis

The selection of critical parameters, such as the order of the fractional derivatives (α) and mask size, plays a crucial role in the performance of the proposed method. Below, we outline the selection process and provide guidelines for optimal settings.

- **Fractional Order (α):** The fractional order controls the extent of differentiation, with lower values (α) favoring smooth regions and higher values (α→) enhancing edges and textures. Optimal α values depend on image characteristics:
  - α=0.4 − 0.6: Suitable for high-noise images (e.g., ultrasound).
  - α=0.5 − 0.7: Suitable for medium-contrast images (e.g., CT).
  - α=0.6 − 0.8: Suitable for high-contrast images (e.g., MRI).
- **Mask Size:** Smaller masks (3x3) are effective for preserving fine details and are computationally efficient, while larger masks (5x5) enhance broader structures but may smooth out finer details. Guidelines include:
  - 3×3 masks: Best for images requiring preservation of fine details (e.g., bone fractures in X-rays).
  - 5×5 masks: Best for images requiring enhanced texture and broader structure emphasis (e.g., soft tissues in CT).

**Sensitivity Analysis:** We conducted a sensitivity analysis to evaluate how variations in the parameters impact the enhancement quality, measured using PSNR, SSIM, and CNR metrics.

1. **Impact of Fractional Order (α):** A range of α values (0.3 to 0.9) was tested on different modalities.
  - **MRI:** α=0.5 − 0.7 provided optimal results, with PSNR increasing by 15% and SSIM by 20%. α<0.4 resulted in oversmoothing, while α >0.8 introduced excessive noise.
  - **CT:** α =0.6 yielded the best balance between detail enhancement and noise suppression, with CNR improving by 18%.
  - **Ultrasound:** α=0.4 −0.5 performed well, effectively reducing speckle noise while preserving textures.
2. **Impact of Mask Size:** The mask size influences both enhancement quality and computational efficiency.
  - Larger masks (5×5) improved CNR by up to 20% for CT and MRI but caused slight over smoothing in ultrasound images.
  - Smaller masks (3×3) maintained sharpness and fine details in X-ray and ultrasound images, with an average SSIM improvement of 15%.

**Guidelines for Optimal Settings:**

- Use α =0.5−0.6 and 3x3 masks for high-noise or low-detail images (e.g., ultrasound).
- Use α =0.6 −0.7 and 5x5 masks for high-contrast or medium-detail images (e.g., CT, MRI).
- Dynamically adjust α based on local image features for images with varying contrast and texture.

## 6 Conclusion

In this study, we introduced a novel approach to enhance single-channel medical images using fractional derivatives, specifically utilizing the Grünwald–Letnikov (GL) fractional differential mask. Our proposed method effectively addresses the limitations of traditional image enhancement techniques by dynamically adjusting the fractional order for each pixel, thereby preserving intricate textures and enhancing image details without over-enhancement. The experimental results demonstrate that the proposed method significantly improves texture enhancement and edge detection in medical images compared to classical methods. The variable and non-linear filter properties of the GL fractional differential mask allow for adaptive enhancement based on specific image features, resulting in better preservation of smooth regions while accentuating important textures and edges. Furthermore, the use of fractional derivatives such as the Atangana-Baleanu derivative in the Caputo sense has proven to be effective in maintaining the integrity of medical images by preserving crucial features and minimizing artifacts. The integration of the Mittag-Leffler function enhances the adaptability of the fractional derivative, providing more precise texture enhancement. Overall, our method shows promising results in medical image processing, offering a more flexible and accurate framework for image enhancement. Future work could explore the application of this technique to other types of images and investigate further improvements in computational efficiency.
